# Occludin Is Involved in Adhesion, Apoptosis, Differentiation and Ca^2+^-Homeostasis of Human Keratinocytes: Implications for Tumorigenesis

**DOI:** 10.1371/journal.pone.0055116

**Published:** 2013-02-04

**Authors:** Susanne Rachow, Michaela Zorn-Kruppa, Ulrich Ohnemus, Nina Kirschner, Sabine Vidal-y-Sy, Peter von den Driesch, Christian Börnchen, Jürgen Eberle, Michael Mildner, Eik Vettorazzi, Rita Rosenthal, Ingrid Moll, Johanna M. Brandner

**Affiliations:** 1 Department of Dermatology and Venerology, University Hospital Hamburg-Eppendorf, Hamburg, Germany; 2 Tabea Clinics GmbH, Hamburg, Germany; 3 Dermatological Center, Klinikum Stuttgart, Stuttgart, Germany; 4 Department of Dermatology, Venerology and Allergology, Skin Cancer Center, Charité, Berlin, Germany; 5 Department of Dermatology, Medical University of Vienna, Vienna, Austria; 6 Department of Medical Biometry and Epidemiology, University Hospital Hamburg-Eppendorf, Hamburg, Germany; 7 Institute of Clinical Physiology, Charité-Universitätsmedizin Berlin, Berlin, Germany; CNRS-University of Toulouse, France

## Abstract

Tight junction (TJ) proteins are involved in a number of cellular functions, including paracellular barrier formation, cell polarization, differentiation, and proliferation. Altered expression of TJ proteins was reported in various epithelial tumors. Here, we used tissue samples of human cutaneous squamous cell carcinoma (SCC), its precursor tumors, as well as sun-exposed and non-sun-exposed skin as a model system to investigate TJ protein alteration at various stages of tumorigenesis. We identified that a broader localization of zonula occludens protein (ZO)-1 and claudin-4 (Cldn-4) as well as downregulation of Cldn-1 in deeper epidermal layers is a frequent event in all the tumor entities as well as in sun-exposed skin, suggesting that these changes result from chronic UV irradiation. In contrast, SCC could be distinguished from the precursor tumors and sun-exposed skin by a frequent complete loss of occludin (Ocln). To elucidate the impact of down-regulation of Ocln, we performed Ocln siRNA experiments in human keratinocytes and uncovered that Ocln downregulation results in decreased epithelial cell-cell adhesion and reduced susceptibility to apoptosis induction by UVB or TNF-related apoptosis-inducing ligand (TRAIL), cellular characteristics for tumorigenesis. Furthermore, an influence on epidermal differentiation was observed, while there was no change of E-cadherin and vimentin, markers for epithelial-mesenchymal transition. Ocln knock-down altered Ca^2+^-homeostasis which may contribute to alterations of cell-cell adhesion and differentiation. As downregulation of Ocln is also seen in SCC derived from other tissues, as well as in other carcinomas, we suggest this as a common principle in tumor pathogenesis, which may be used as a target for therapeutic intervention.

## Introduction

Tight Junctions (TJs) are multiprotein complexes formed by transmembrane proteins, e.g. occludin (Ocln), claudins (Cldns), and junctional adhesion molecules (JAMs), which are associated with intracellular plaque proteins, e.g. ZO-1, 2 and 3 and MUPP-1. From simple epithelia it is known that TJs seal neighbouring cells and control the paracellular pathway for solutes, water, and cells (barrier function). In addition, they restrict the diffusion of apical and basolateral membrane components (fence function), coordinate signalling molecules and play a role in cell differentiation and proliferation [1], [2], [3], [4], [5], [6]. A role of TJs in paracellular barrier function was also shown in the multi-layered epithelium of the skin [7], [8], and an involvement of Cldn-1 in proliferation and differentiation of keratinocytes was suggested [9], [10], [11].

There are frequent changes in TJ protein localization and/or expression in the course of carcinogenesis. For Cldns, up- or downregulation, as well as altered localization were described, dependent on the tumor entity. For instance, in breast cancer Cldn-1, -2, and -7 are downregulated, while Cldn-4 is upregulated and in colorectal- and pancreatic-cancer Cldn-1, -2, and -7 are upregulated [12], [13], [14], [15], [16]. For Ocln, mainly a downregulation was observed in various tumors [17], [18], [19], [20]. Downregulation of Ocln is a common feature of epithelial-mesenchymal-transition (EMT) in tumors derived from simple epithelial cells, and regulation of Ocln was described by the oncogenic Raf1 pathway as well as by the transcription factors slug, and snail [21], [22], [23], [24], [25]. Furthermore, an involvement of Ocln in apoptosis has been suggested, but there is conflicting evidence whether apoptosis is enhanced or suppressed (see also discussion) [26], [27], [28], [29], [30]. For ZO-1 down- or upregulation was observed in different tumor entities [17], [31], [32], and its redistribution from cell-cell-borders to the cytoplasm and nucleus was described in EMT [e.g. 33]. Decreased expression of TJ proteins suggests that tumorigenesis is accompanied by TJ disruption and loss of cell-cell adhesion followed by loss of differentiation, uncontrolled proliferation, and invasiveness as well as increased supply with nutrition. However, up-regulation of TJ proteins may also be an initial step which disturbs the balance of TJs and therefore cell homeostasis [12], [15], [34].

Squamous cell carcinoma (SCC) is the second most common skin malignancy accounting for the majority of non-melanoma skin cancer-related metastatic disease and death [35]. It can be categorized in well, moderately and poorly differentiated SCCs [36], [37]. Epidermal differentiation marker involucrin was described to be present in lower malignant, well differentiated SCCs but to be decreased in higher malignant, poorly differentiated ones [38], [39], [40], Transglutaminase 1 (TG1) was described to be increased in the epidermal part of the SCCs but is absent in invasive parts [41]. Actinic keratoses (AK) and Bowen’s disease (BD) are suggested to be precursors (c*arcinomata in situ*) of SCC and their presence is a marker for increased risk for the occurrence of SCC [42]. Keratoacanthoma (KA) represents a closely related epithelial tumor and can transform to SCC especially in elderly persons [37]. SCC and its precursors mainly develop as the result of UV damage on chronically sun-exposed skin. Due to their good accessibility, SCC and its precursors are a good model system to investigate tumor progression from normal (non-sun-exposed skin) via stressed (sun-exposed skin) multi-layered epithelia to in-situ (AK, BD), low malignant (KA) and high malignant (SCC) carcinoma.

There is limited data about TJ protein alterations in human cutaneous SCC. Langbein et al. [43] investigated three cases and found punctate or extended cell-cell border structures positive for Ocln, Cldn-1, Cldn-4, cingulin, and ZO-1 in higher as well as in lower differentiated areas. On the other hand, Morita et al. [44] described a restriction of strong expression of Cldn-1, Cldn-4, Ocln, and ZO-1 to keratinized areas in 5 SCC. In unkeratinized tumor cells, Cldn-1 was heterogeneously expressed, ZO-1 was weak, whereas Ocln and Cldn-4 were absent. In 5 cases of BD, no loss of Ocln and Cldn-4 but an aberrant localization were seen [44].

These interesting but somewhat discrepant findings in only a limited number of SCC and BD cases and the putative impact of TJ protein alterations on tumor progression prompted us to investigate larger numbers of cutaneous SCC and their precursors including (chronically) sun-exposed skin, as well as healthy, non-sun-exposed skin with the aim to identify similarities and differences for TJ proteins. Because we observed a striking loss of Ocln in many malignant SCC in contrast to its precursors we hypothesized that this molecule might promote tumorigenic features in keratinocytes which was addressed by investigating the involvement of Ocln in cell-cell adhesion, apoptosis, proliferation, differentiation and Ca^2+^-homeostasis in knock-down studies.

## Materials and Methods

### Ethics Statement

The samples for this study were obtained from our clinical department. They were used after diagnostic procedures had been completed. The local medical ethics committee (Aerztekammer Hamburg) approved this study (060900 and OB-008/04). All patients gave their written informed consent.

### Tissues, Cells, Antibodies, qPCR Primers and siRNAs

Samples of human SCC (n = 46; 31 male, 15 female; age 19–93 y, mean: 73 y), BD (n = 26; 15 male, 11 female; age 33–94 y, mean 74 y), KA (n = 24; 15 male, 9 female; age 21–89 y; mean 59 y), AK (n = 25, 12 male, 13 female, age 61–92 y, mean 72 y) sun-exposed skin (n = 11, 4 male, 7 female; age 16–89 y, mean: 67 y) and non-sun-exposed skin (n = 17, 9 male, 8 female; age 20–78 y; mean 42 y) were obtained from our clinical department. Grading of the SCC was performed according to the world health organisation classification [37]. Human foreskin keratinocytes were isolated and cultured as described [45]. Antibodies used are summarized in Table 1. FAM™ dye-labeled real-time PCR (qRT-PCR) TaqMan® MGB probes for Ocln (Hs00170162_m1), involucrin (Hs0084307_s1), TG1 (Hs01070310_m1), Cldn-2 (Hs00252666_s1), Cldn-12 (Hs00221623_m1) and GAPDH (Hs03929097_g1) were purchased from Applied Biosystems (Carlsbad, CA, USA). Ready-to-use siRNAs for human Ocln (Hs_OCLN_7: SI03225999, Hs_OCLN_9: SI04360034), ZO-1 (SI02655149), and control siRNA (1027280) were purchased from QIAGEN (Hilden, Germany). Stealth siRNAs for gene silencing of Ocln in human organotypic skin models [46] were purchased from Life Technologies (Darmstadt, Germany; siRNA1: HSS107401; siRNA3: HSS181629; control: 12935–300). Due to technical reasons different siRNAs had to be used for cell culture and skin model experiments.

**Table 1:** Antibodies and nuclear dyes.

Target	Clone/lot	Company	Dilution IF	Dilution Western-blot
**Cldn-1**	2H10D10	Zymed Laboratories	1∶150	
**Cldn-4**	3E2Cl	Zymed Laboratories	1∶50	
**Zo-1**	1A12	Zymed Laboratories	1∶80	
**Ocln**	OC-3F1071–1500	Zymed Laboratories	1∶80	1∶2000
**JAM-A**	Rm-JAM-1	R&D systems	1∶30	
**TG1**	BC1	Antibody online	1∶20	1∶500
**Involucrin**	SY5	Novo Castra	1∶200	1∶2000, pH 8.0
**Ki67**	MIB-1	DAKO	1∶50	
**E-Cadherin**	NCH-38	DAKO	1∶50	1∶100
**Vimentin**	GP53	Progen	1∶1000	1∶500
**Actin**	AC-15	Sigma		1∶10000
**Tubulin**	DM-1A	Calbiochem		1∶2000
**Nuclei**	DAPI	Boehringer Mannheim	1∶5000	

Abbreviations: Cldn, claudin; JAM, junctional adhesion molecule; IF, immuno-fluorescence; Ocln, occludin; TG1, transglutaminase 1; ZO, zonula occludens.

IF treatment for TG1: Citrate buffer, 2×10 min microwave, 0.001% trypsin, DAKO Block overnight. IF treatment for all others: TEC buffer, 2×10 min microwave, 0.001% trypsin, DAKO block.

### Immunofluorescence Microscopy

**Figure 1:**
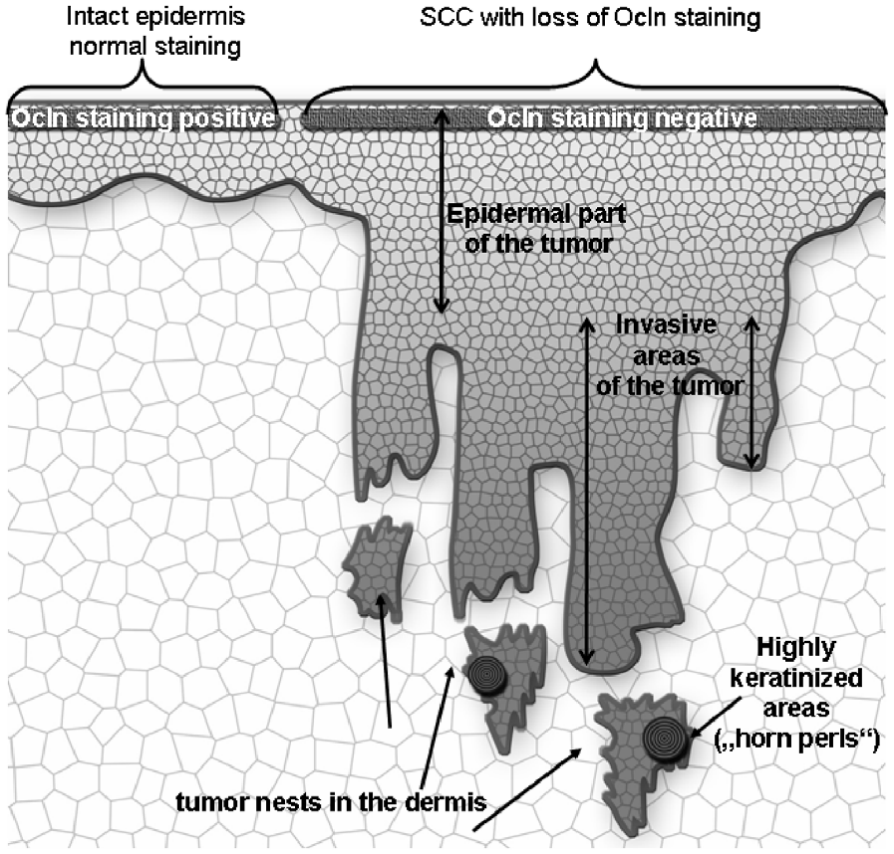
Schematic drawing of a squamous cell carcinoma denoting the different areas of the tumor.

Immunofluorescence microscopy was performed as previously described [45]. For details see Table 1. An Axiophot-II microscope (Carl Zeiss; Jena/Oberkochen, Germany), a CCD-Camera (Hamamatsu Photonics; Hamamatsu City, Japan), and Openlab 2.0.9 software (Improvision; Coventry, UK) were utilized to visualize and evaluate the stained sections. ZO-1 and especially Ocln are very sensitive antigens influenced by slight deviations of fixation. Therefore samples were only evaluated when positive staining in the “uninvolved epidermis” (Figure 1) and/or internal positive controls (e.g. sweat glands) confirmed the success of staining procedure and stainings were repeated at least twice. The stainings were analyzed by two independent investigators (SR and JMB). The number of evaluated samples was as follows: SCC (Ocln: n = 35, ZO-1: n = 32, Cldn-1, JAM-A: n = 46; Cldn-4: n = 30, Inv: n = 41, TG1: n = 11; E-cadherin: n = 12; vimentin: n = 10 ), BD (Ocln: n = 19, ZO-1: n = 25, Cldn-1: n = 25, JAM-A: n = 26; Cldn-4: not tested), KA (Ocln: n = 18, ZO-1: n = 23, Cldn-1, JAM-A: n = 24; Cldn-4: not tested), AK (Ocln: n = 19, ZO-1: n = 22; Cldn-1: n = 24; JAM-1: n = 25; Cldn-4: n = 9), sun-exposed skin (Ocln: n = 9, ZO-1: n = 9; Cldn-1: n = 10; JAM-A: n = 9; Cldn-4: n = 11), non-sun-exposed healthy skin (Ocln: n = 11, ZO-1: n = 15; Cldn-1: n = 16; JAM-A: n = 15; Cldn-4: n = 15).

### Human Organotypic Skin Models (3D Models)

Pre-confluent human primary keratinocytes were transfected with the respective siRNA using Lipofectamine 2000 (Invitrogen) according to the manufacturer’s instructions. Keratinocytes were then incubated for 24 h before seeding onto a fibroblast collagen gel as described below.

Organotypic skin models were prepared as described previously [47]. Briefly, a 2.5 ml suspension of collagen type I (Advanced Biomatrix, San Diego, CA, USA) containing 2.5×10^5^ fibroblasts was poured into cell-culture inserts (3 µm pore size; BD Bioscience, Bedford, MA, USA) and allowed to gel for 2 h at 37°C without CO_2_. The gels were then equilibrated with KGM medium (Lonza, Basel, Switzerland) at 37°C in a CO_2_ incubator for 2 h, and 1.5×10^6^ keratinocytes, transfected with the respective siRNAs, were seeded onto each collagen gel. After overnight incubation at 37°C the medium was removed from both the inserts and external wells, and 10 ml serum-free keratinocyte defined medium (SKDM), consisting of KGM without bovine pituitary extract and supplemented with 1.3 mM calcium (Sigma, Vienna, Austria), 10 µg/ml transferrin (Sigma), 50 µg/ml ascorbic acid (Sigma), and 0.1% bovine serum albumin (Sigma), was added to each external well. The organotypic skin models were cultured for 7 days and medium was changed every second day.

### siRNA Experiments

Primary human keratinocytes were transfected under low Ca^2+^ conditions by using HiPerFect Transfection reagent (Qiagen, Hilden, Germany) according to the manufacturer’s instructions. Briefly, cells were transfected by a fast forward protocol. 100000 cells/ml were transfected with a 1∶1 mixture of HiPerFect reagent (1∶200) and 100 nM of Ocln-siRNA or control-siRNA, respectively. Efficiency of knock-down was controlled by Western-blot and qRT-PCR analysis after 2, 3 or 5 days (depending on the experiment). Silencing of Ocln in a human organotypic skin model was performed as described [46], [47]. Briefly, transfection was performed using Lipofectamine 2000 (Invitrogen) according to the manufacturer’s instructions with slight modifications. Third passage keratinocytes were grown to 70–80% confluency. Lipofectamine 2000 (50 µl) was mixed with 100 µl of a 20 µM siRNA solution and 5 ml OPTI-MEM medium (Gibco). After 30 min at room temperature, KGM (20 ml) was added and the solution was poured onto the keratinocytes monolayer (25 ml) for 24 h. After transfection keratinocytes were trypsinized and seeded onto a fibroblast collagen gel. Efficiency of knock-down was controlled by Western-blot and qRT-PCR analysis.

### BrdU-assay

Proliferation experiments under low Ca^2+^ conditions were performed 48 h after seeding of 8000 cells/well and siRNA-treatment. Subsequently, Ca^2+^ levels were elevated to 2 mM to induce differentiation and 48 h later proliferation experiments were performed under high Ca^2+^ conditions. The BrdU-ELISA kit from Roche (Mannheim, Germany) was used following manufacturer’s instructions.

### Evaluation of Proliferative Cells in 3D Models

Proliferative (Ki67-positive) cells were stained by MIB-1 and at least 5 visual fields (0.25 mm^2^) were evaluated per model (n = 4). In each visual field the number of Ki67 positive cells was normalized to the total number of basal cells which were evaluated by DAPI staining.

### Cell Adhesion Assays

Intercellular adhesion under high Ca^2+^ conditions was tested using a hanging drop assay which was modified from [48]. Briefly, following 48 h of siRNA silencing cell suspensions of 550000 single cells per ml were prepared. 35 µl drops of the suspensions were placed under the lids of 60×15 mm tissue culture dishes. To limit evaporation 2 ml of PBS was added to the bottoms of the dishes. After indicated time points the drops were pipetted and placed in an improved Neubauer hemocytometer chamber. Pictures of 4×12 Neubauer-squares of 0.0625 mm^2^ each were taken using a Leica DM LS microscope with 10× objective magnification and a Leica EC3 digital camera (*Leica* Biosystems *Nussloch* GmbH, *Nussloch*, Germany). The numbers of particles in the squares were determined automatically by utilizing FIJI software [49] and the particles analyzer tool. The relative cell adhesion activity was evaluated according to [50] by calculating the cell aggregation index Nt/N0×100, where Nt is the number of particles at different time points and N0 the initial particle number. Lower numbers of particles reflect increased cell-cell adhesion.

### TRAIL Treatment and UVB-irradiation

For TRAIL treatment and UVB-irradiation, keratinocytes were plated in 96-well plates (8000 cells/well) under low Ca^2+^ conditions and transfected for 48 h with the indicated siRNAs. Then cells were transferred to siRNA-free medium for another 24 h and subsequently exposed to TRAIL (25 ng/ml; Axxorra/Alexis Gruenberg, Germany) or irradiated with 150 mJ/cm^2^ to induce apoptosis. Irradiation was performed in PBS, and then the solution was replaced by fresh medium.

### Apoptosis and LDH Assay

Apoptosis was evaluated at 3 h and 20 h of TRAIL treatment and at 16 h after UVB-irradiation by using the Cell Death Detection ELISA^PLUS^ kit (Roche) according to the manufacturer’s instructions. This assay is defined as release of mono- and oligonucleosomes due to DNA fragmentation in the cytoplasm of apoptotic cells. Cytotoxicity was determined after 3 h and 20 h of TRAIL treatment as well as 4 h and 16 h after UVB irradiation by a lactate dehydrogenase (LDH) release assay (Cytotoxicity Detection kit^PLUS^ - LDH; Roche). Relative apoptosis was calculated by dividing the absorbance of treated cells by the absorbance of non-transfected non TRAIL-or UV-treated control cells. The percentages of cytotoxicity were calculated according to a control with completely lysed cells due to lysis buffer treatment.

### Analysis of Ca^2+^ Permeability

Determination of Ca^2+^ permeability was performed in Ussing chambers specially designed for the investigation of cell monolayers grown on culture plate inserts [51]. Keratinocytes were seeded on culture plate inserts (Millicell-PCF, pore size 0.4 µm, area 0.6 cm^2^; Millipore), cultured under high Ca^2+^-conditions for 48 h, and investigated in the Ussing chambers. Ca^2+^ permeability was determined from biionic potentials and the Goldman–Hodgkin–Katz equation as reported in detail previously [52].

### Statistical Analysis

Statistical significance of proliferation, adhesion, Ca^2+^-barrier-formation and apoptosis was determined by Student’s t-test. TJ protein expressions were cross-tabulated with tumor grading, differentiation markers, and sun-exposure and the significance of associations were tested using Fisher’s Exact Test. P-values <0.05 were considered as statistically significant (* p<0.05, ** p<0.01, *** p<0.001).

## Results

### Aberrant Localization Patterns of TJ Proteins in Cutaneous SCC

**Figure 2:**
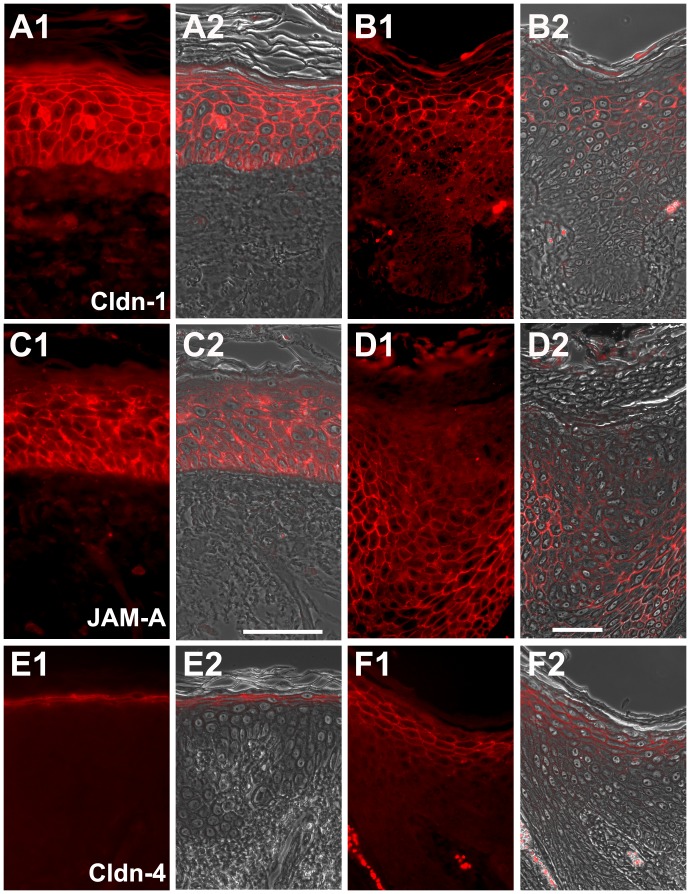
Immunolocalization of Cldn-1, JAM-A, and Cldn-4 in healthy, non-sun- exposed skin and SCC. Immunolocalization of Cldn-1 (A1, A2, B1, B2), JAM-A (C1, C2, D1, D2), and Cldn-4 (E1, E2, F1, F2) in healthy, non-sun-exposed skin (A1, A2, C1, C2, E1, E2) and SCC (B1, B2, D1, D2, F1, F2). (A1, B1, C1, D1, E1, F1: epifluorescence pictures; A2, B2, C2, D2, E2, F2: overlay of epifluorescence and phase contrast pictures). Note that Cldn-1 is downregulated in the uppermost and lowermost layers whereas JAM-A is primarily downregulated in the uppermost layers of SCC. Furthermore Cldn-4 demonstrates broader localization in SCC compared to healthy skin. Bars: 50 µm.

**Figure 3:**
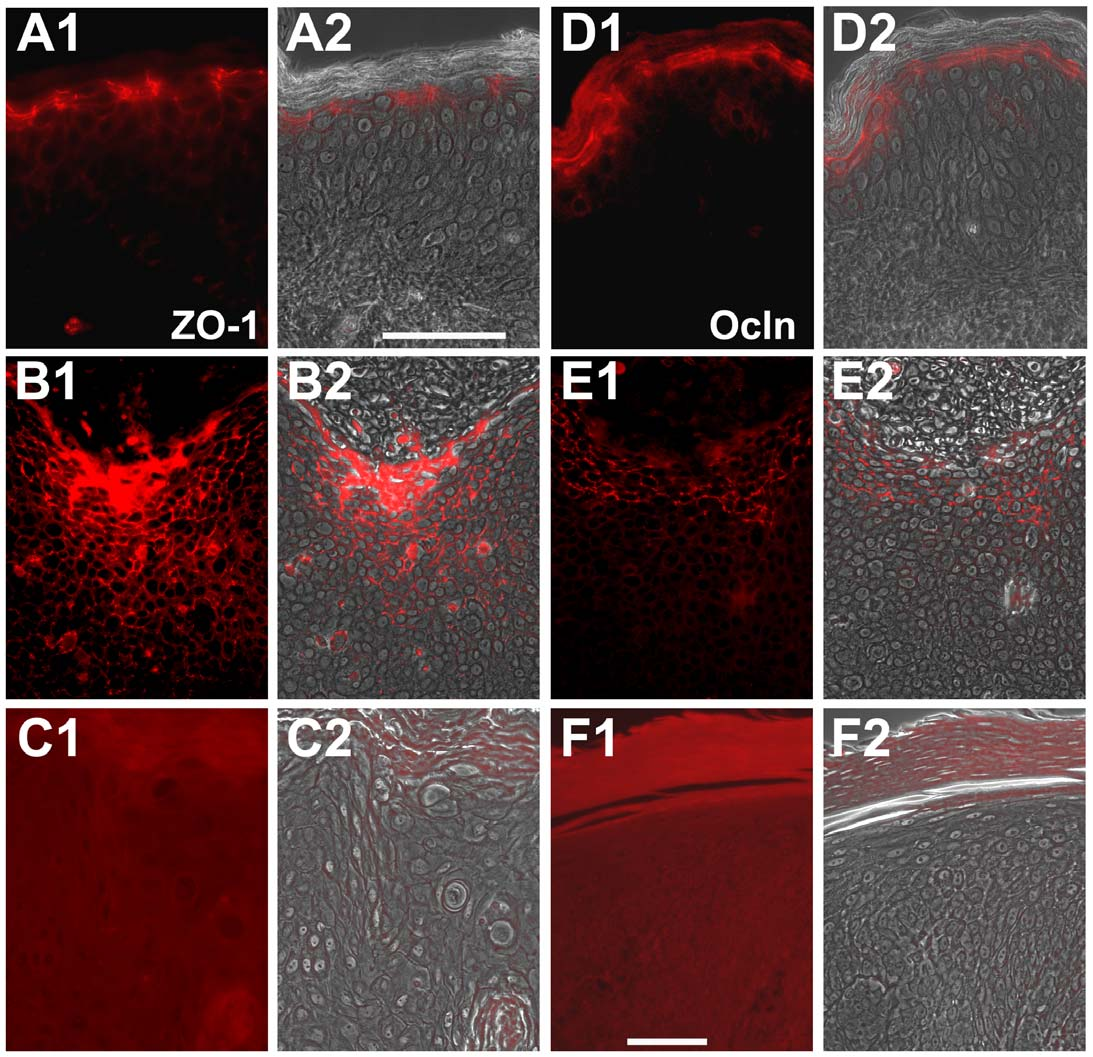
Immunolocalization of ZO-1 and Ocln in healthy, non-sun-exposed skin and SCC. Immunolocalization of ZO-1 (A1, A2, B1, B2, C1, C2), and Ocln (D1, D2, E1, E2, F1, F2), in healthy, non-sun-exposed skin (A1, A2, D1, D2) and SCC (B1, B2, C1, C2, E1, E2, F1, F2). (A1, B1, C1, D1, E1, F1: epifluorescence pictures; A2, B2, C2, D2, E2, F2: overlay of epifluorescence and phase contrast pictures). For both molecules an example of broader localization (B1, B2, E1, E2) and loss of the protein (C1, C2, F1, F2) are shown. Note that exposure time in C1, C2, F1, F2 was substantially higher than in the other figures in order to show that also with very high exposure time no Ocln or ZO-1 staining was seen in these samples. Bars: 50 µm.

In normal, non-sun-exposed skin Cldn-1 and JAM-A were predominantly found at the cell-cell borders of all living epidermal layers, while localization of Cldn-4, Ocln and ZO-1 was mainly restricted to cell-cell borders of the stratum granulosum (Figure 2, 3). In addition, cytoplasmic staining was observed (Figure 2, 3).

In contrast, in SCC samples there was a complete loss of staining for ZO-1 in 25% of the tumors, for Ocln in 80% (Figure 3), for Cldn-1 in 9%, and for Cldn-4 in 17% (Table 2). Also in samples positive for these proteins there were some negative spots (data not shown). No cases were completely negative for JAM-A.

**Table 2:** Protein expression and localization of TJ proteins in the various skin tumors.

	Squamous cell carcinoma		Kerato-acanthoma		Bowens Disease		Actinic keratoses	
	Loss	Altered	Loss	Altered	Loss	Altered	Loss	Altered
**Cldn-1**	9%	60%^1^/81%^2^	0%	68%^1^/80%^2^	4%	55%^1^/86%^2^	4%	83%^1^/83%^2^
**Cldn-4**	17%	91%^3^	Not tested	Not tested	Not tested	Not tested	0%	91%^3^
**JAM-A**	0%	85%^1^	0%	88%^1^	0%	78%^1^	4%	73%^1^
**Ocln**	80%	29%^3^	29%	71%^3^	11%	42%^3^	5%	54%^3^
**ZO-1**	25%	67%^3^	0%	88%^3^	12%	65%^3^	9%	75%^3^

Loss: complete loss of expression, altered: altered localization. % in “loss of expression” denotes the percentage of all tumors, % in “altered localization” denotes the percentage of positive tumors.

1 downregulation in the uppermost layers.

2 downregulation in the lowermost layers.

3 broader expression.

Abbreviations see table 1.

In 81% of the positive cases for Cldn-1, there was a downregulation of the protein in the lowermost and in 60% in the uppermost layers of epidermal tumor parts (Figure 1, 2, Table 2). JAM-A was downregulated in the upper layers in 85% of the samples (Figure 2, Table 2). Cldn-4 showed a broader localization in 91% of the samples (Figure 2), ZO-1 in 67%, and Ocln in 29% (Figure 3, Table 2).

In the invasive parts of the tumors (Figure 1), Ocln and Cldn-4 were absent, Cldn-1 was observed in one and ZO-1 in two cases. JAM-A was present in all cases (data not shown).

This indicates that complete loss of Ocln and downregulation of Cldn-1 in the lowermost and JAM-A and Cldn-1 in the uppermost layers is a frequent feature in SCC. This also applies for the broader localization of Cldn-4 and ZO-1.

### Localization Patterns of TJ Proteins in AK, BD, and KA

We wondered whether the alterations found in SCC may also be seen in in-situ carcinomas (AK, BD) or in tumors of lower malignancy (KA).

Complete loss of Ocln was found in only 5% of AK, 11% of BD, and 29% of KA, demonstrating that its absence is much more characteristic for SCC (80%). Contrariwise, broader localization of Ocln was found more frequently in the precursor tumors (42–71%, as compared to 29%; Table 2).

For ZO-1, complete loss was observed in 9% of AK, 12% of BD but not in KA (25% in SCC). Frequency of broader localization was similar between the tumor entities. Furthermore, there was no clear-cut difference between SCC and the other tumors as concerning Cldn-1, Cldn-4, and JAM-A (Table 2). Similar to SCC samples, we observed also in the SCC precursor samples spots with lacking expression of TJ proteins.

The data indicate that alterations found for Cldn-1, Cldn-4, ZO-1, and JAM-A are common for UV-induced skin tumors while the frequent loss of Ocln appears as specific for SCC.

### Localization Patterns of TJ Proteins in Sun-exposed Versus Non-sun-exposed Skin

**Figure 4:**
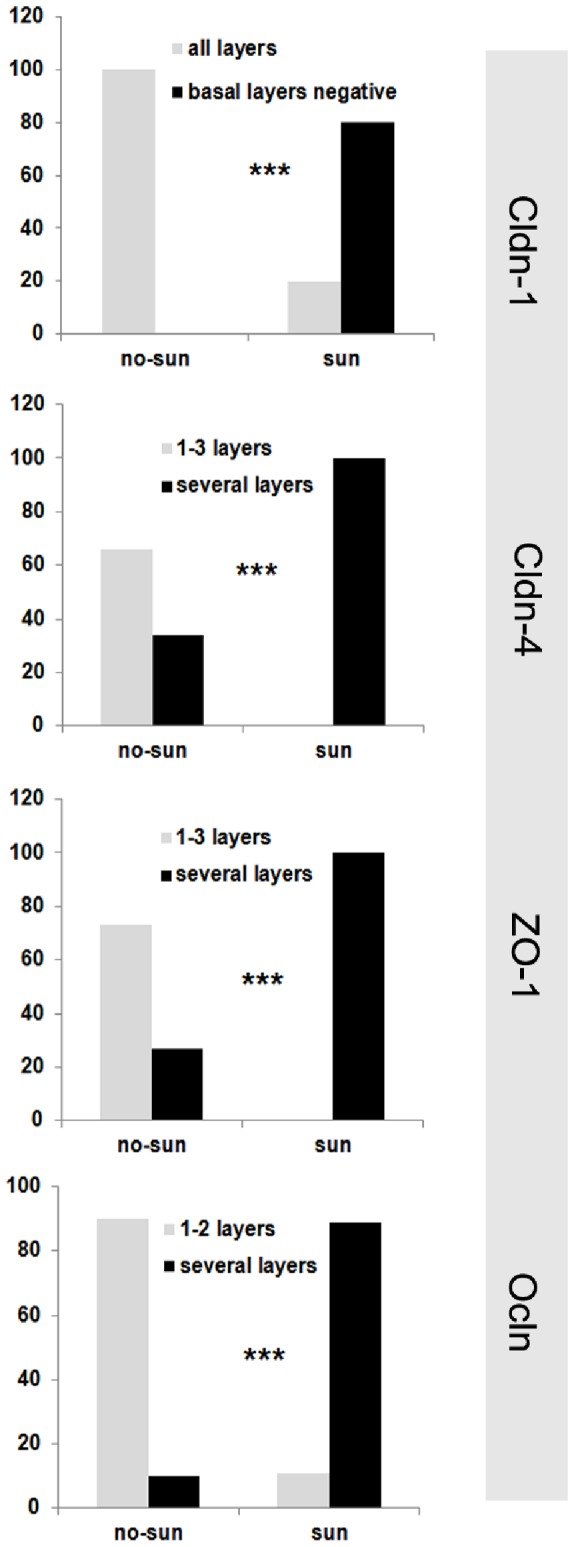
Distribution patterns of TJ proteins in normal skin from sun-exposed and non-sun-exposed areas. Percentages denote distribution patterns of the various TJ proteins in sun-exposed (sun; e. g. face, lower arms) and non-sun-exposed areas (no-sun; e. g. abdomen, bottom). ***: p<0.001 between sun-exposed and non-sun-exposed skin.

Because the alterations for Cldn-1, Cldn-4, and ZO-1 were observed in all UV- promoted skin tumors and a broader localization of Ocln was frequent in all tumor entities except for SCC, we wondered whether these alterations could also be identified in chronically sun-exposed skin. Indeed, a broader localization of Ocln, ZO-1, and Cldn-4 was seen more frequent in the epidermis of sun-exposed as compared to non-sun-exposed skin with high statistical significance (Ocln: p = 0.001, ZO-1: p = 0.001, Cldn-4: p = 0.001) (Figure 4). We found no significant influence of age and sex on TJ protein localization. However, a complete loss of Ocln, as in SCC, was not observed in sun-exposed skin. Also spots with loss of staining were less frequent than in the tumors (data not shown). For Cldn-1, we observed a downregulation in the lowermost epidermal layers in sun-exposed skin which was less frequent in non-sun-exposed skin (p<0.001) (Figure 4). Downregulation in the uppermost layers - as frequently seen in the tumors - was not observed. From this data we conclude that most TJ protein alterations observed in skin tumors are likely to be induced by UV-irradiation, but loss of Ocln and downregulation of Cldn-1 in the uppermost layers may not be related to UV.

### Knock-down of Occludin Alters Epidermal Differentiation Markers but not Markers for EMT

**Figure 5:**
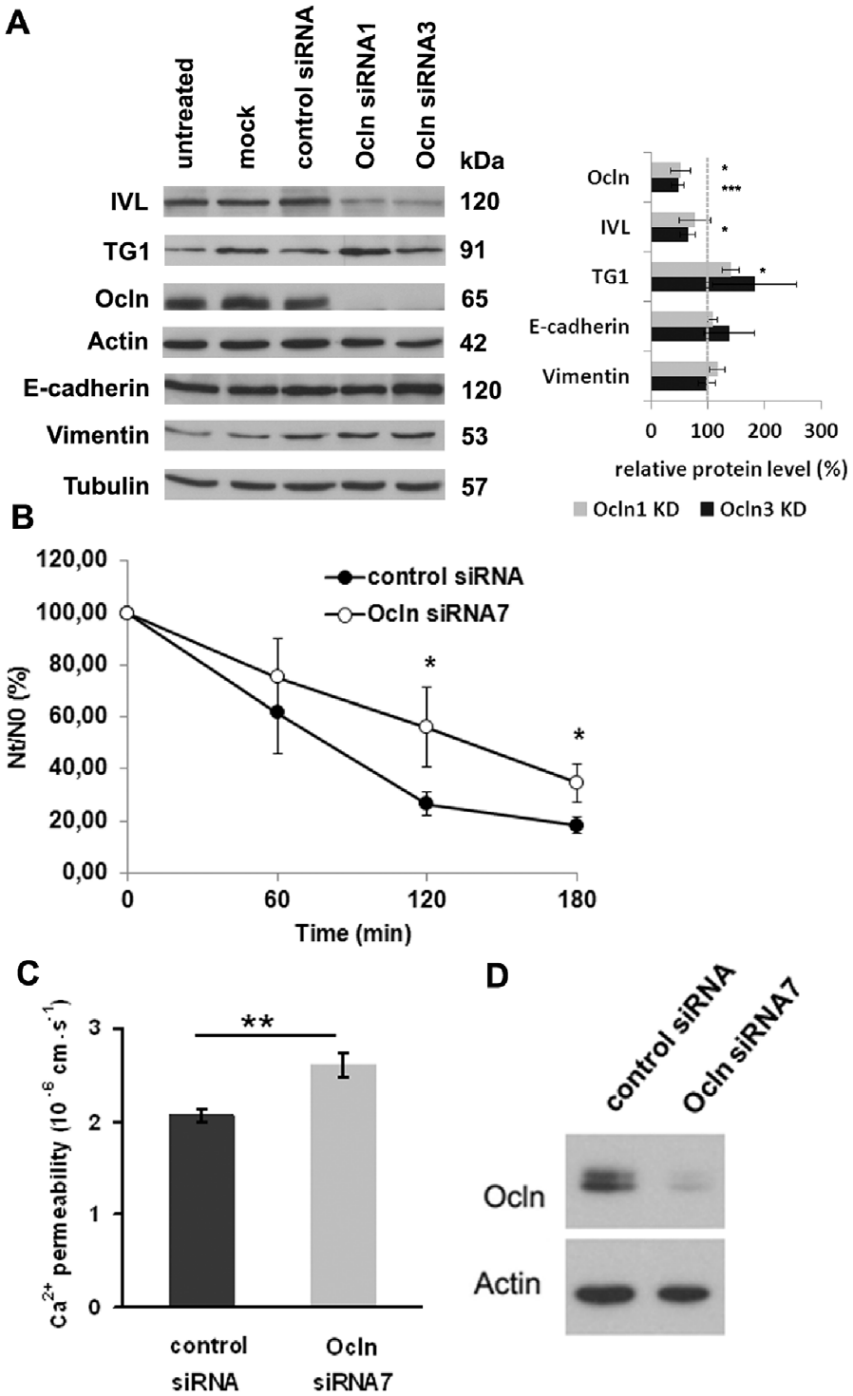
Influence of Ocln on epidermal differentiation and EMT markers, cell-cell adhesion and paracellular Ca^2+^ permeability. (A) (left side) Western Blot analysis of 3D skin models after silencing of Ocln with 2 different siRNAs shows downregulation of Ocln and of the epidermal differentiation marker involucrin and an upregulation of TG1. There is no alteration for the EMT markers E-cadherin and vimentin. Same amounts of protein were loaded and actin or tubulin were used as gel loading controls. A representative experiment is shown (n = 3). (right side) Semiquantitative analysis of Ocln, involucrin, TG1, E-cadherin and vimentin. Band intensities were normalized to actin (Inv, TG1) or tubulin (E-cad, vim). Subsequently, the values were normalized to control siRNA treated cells (n = 3; mean±SEM; *p<0.05, ***p<0.001 compared to control siRNA). (B) Calcium induced cell-cell adhesion was investigated with a hanging drop assay at the indicated time points. Significantly greater numbers of particles (indicating less cell-cell adhesion) were found in the suspensions of Ocln knock-down cells compared to the controls (*: *p*<0.05 *n* = 3), (C) Electrophysiological studies of paracellular Ca^2+^-permeability in Ocln siRNA-treated cultured keratinocytes revealed an increase in paracellular permeability for Ca^2+^ in Ocln knock-down cells compared to cells treated with control siRNA (n = 6, mean ± SEM). (D) Example for the knock-down of Ocln in siRNA treated submerged cells. Mean knock-down of Ocln in the cells used for experiments in Figure 5 B, C and 6 was 76% +/−9%.

Altered differentiation is a common feature in tumor progression. Accordingly, irregularity of epidermal differentiation markers involucrin and TG1 was described in SCC [38], [39], [40]. Therefore we investigated whether downregulation of Ocln may influence epidermal differentiation. When using two different siRNAs in a 3D skin model, we could strongly downregulate Ocln protein expression (Figure 5A). This was associated with downregulation of the differentiation marker involucrin and an upregulation of TG1 (Figure 5A). The same effect was seen for involucrin when investigating 2D cell cultures (data not shown). On mRNA-level, a slight and partly significant upregulation for involucrin and TG1 was observed (Involucrin: Ocln siRNA1∶1.4±0.4, n.s., Ocln siRNA3∶2.2±0.5, p<0.01; TG1: Ocln siRNA1∶1.4±0.1, p<0.001; Ocln siRNA3∶1.7±0.6, n.s.; Ocln: Ocln siRNA1∶0.4±0.2, p<0.01; Ocln siRNA3∶0.4±0.2, p<0.01; n = 3).

Because Ocln was also described to be involved in epithelial-mesenchymal transition [21], [22], [25], we investigated E-cadherin as an epithelial marker as well as vimentin as a mesenchymal marker for EMT in the Ocln knock-down models. However, we did not find any changes of these markers (Figure 5A).

Further, we looked for correlations between involucrin, and TG1, on one hand and Ocln expression on the other hand in the SCC samples as well as for correlations of Ocln with the SCC grade. We found that Ocln-positive tumors were to a higher percentage differentiated tumors (Grade I; 57.1%) than Ocln-negative tumors (25.9%) (Table 3). Characteristically, all Ocln-positive tumors were also positive for involucrin (Table 4). However, also 84% of Ocln-negative tumors were still positive for involucrin (see Table 3, 4). For TG1, 71% of the Ocln-negative tumors showed an upregulation in the epidermal parts, however, this was also the case in 100% of Ocln-positive tumors (Table 4).

**Table 3:** Correlation of presence of TJ proteins and tumor grading of SCC.

	Ocln		ZO-1		Cldn-1		Cldn-4	
	Pos	Neg	Pos	Neg	Pos	Neg	Pos	Neg
**Tumor grading**								
**I**	57.1%	25.9%	27.3%	25.0%	32.5%	50%	20.8%	40%
**II**	28.6%	40.7%	45.4%	37.5%	42.5%	25%	50%	20%
**III**	14.3%	33.4%	27.3%	37.5%	25%	25%	29.2%	40%

Pos, positive; neg, negative. Further abbreviations see table 1.

**Table 4:** Correlation of the presence of TJ proteins and differentiation markers involucrin and TG1 as well as EMT markers E-cadherin and vimentin in SCC.

	Involucrin		TG1		E-Cadherin		Vimentin	
	Pos	Neg	Normal	Up-regulated	Normal or slightlydown-regu-lated	Strongly down-regulated	Pos	Neg
**Ocln- pos**	100% 7/7	0%0/7	0%0/4	100% 4/4	67% 4/6	23% 2/6	20% 1/5	80% 4/5
**Ocln-neg**	84% 21/25	16% 4/25	29% 2/7	71%5/7	86% 6/7	14% 1/7	0%0/5	100% 5/5

Abbreviations see table 1 and 3. n/m denotes the positive/negative or normal/upregulated cases compared to the total amount of cases of a specific category.

### Knock-down of Occludin Reduces Cell-cell Adhesion

We asked whether the alteration of Ocln might also influence keratinocyte adhesion, because loss of adhesion is also a prerequisite for tumor invasion and metastasis. Indeed, significantly reduced keratinocyte-keratinocyte adhesion was seen in response to Ocln knock-down, as shown by a cell adhesion assay in a time kinetic analysis (Figure 5B, D).

### Knock-down of Occludin Increases Paracellular Permeability for Ca^2+^

Cell-cell adhesion as well as cell-differentiation is dependent on Ca^2+^-homeostasis. Therefore, we investigated whether decreased Ocln levels may also alter Ca^2+^ permeability of keratinocyte-sheets. Indeed, increased paracellular Ca^2+^ permeability (126% ±5%) was observed in Ocln knock-down cells as compared to control-siRNA-treated cells (Figure 5C, D). Consequently, knock-down of Ocln also reduced transepithelial resistance (data not shown). Because Cldn-2 and Cldn-12 are known to be key-players in Ca^2+^-permeability in intestinal cells [53] we investigated the influence of Ocln knock-down on mRNA expression of these claudins. While there was no influence on Cldn-12, we observed a significant downregulation of Cldn-2 to 0.56+/−0.05 of the level of control cells (n = 3).

### Knock-down of Occludin Reduces the Susceptibility of Keratinocytes for Induction of Apoptosis

A tumor-promoting effect may also result from inhibition of apoptosis. We therefore addressed the question whether downregulation of Ocln may also result in a reduced sensitivity of keratinocytes for proapoptotic stimuli. We tested here TRAIL, which plays a particular role in keratinocyte regulation of apoptosis [54], and UVB-irradiation, a typical apoptosis inducing stimulus in the skin.

**Figure 6:**
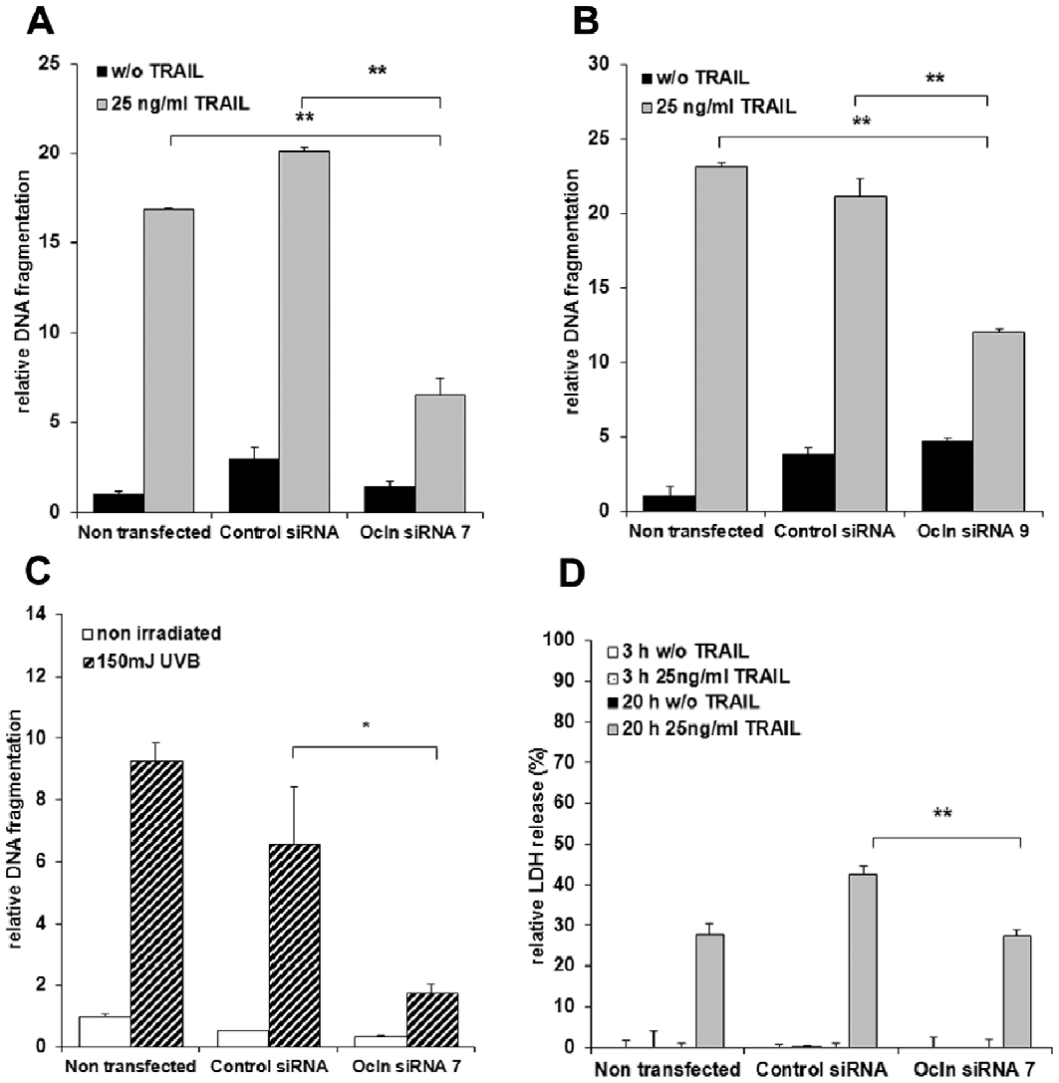
Influence of Ocln on DNA fragmentation after induction of apoptosis by TRAIL and UVB. Apoptotic response at 20 h of TRAIL treatment (25 ng/ml) (A+B) or 16 h after UVB-irradiation (C) in human keratinocyte cultures with or without Ocln knock-down due to Ocln siRNA7 (A+C+D) or Ocln siRNA9 treatment (B). A significant decrease of apoptotic DNA fragmentation induced by TRAIL and UV light was observed after downregulation of Ocln. (D) Cytotoxicity measured by LDH assay at 3 h and 20 h of TRAIL treatment (100% corresponds to complete lysis of the cells). Note that 3 h after TRAIL treatment (when apoptotic fragmentation was already detectable) cytotoxicity was not primarily influenced indicating an apoptotic rather than necrotic mechanism. Mean +/− SEM. *: p<0.05; ** p<0.01. Example of one out of at least 10 independent cell lines. All experiments were performed in triplicates.

Indeed, significantly decreased apoptosis was evident after 3 h (data not shown) and 20 h (Figure 6A, B) of TRAIL-treatment in Ocln knock-down keratinocytes compared to control siRNA-treated cells as seen by using two different siRNAs (Figure 6A, B). In addition, a clear decrease of UVB-induced apoptosis was seen in Ocln knock-down cells (Figure 6C).

Thus, Ocln knock-down selectively prevented apoptosis induction by TRAIL and UVB, which may suggest a role of Ocln loss in tumor progression of SCC, based on apoptosis resistance to different stimuli.

Of note, the effects of Ocln knock-down were not seen in all keratinocyte cultures. The effects on TRAIL-induced apoptosis were seen in 50% of the primary keratinocyte cultures (n = 14) that originated from different donors, and the suppressive effects on UVB-induced apoptosis were seen in 40% of cultures (n = 10). This was independent from the level of Ocln knock-down. All primary cultures that showed an effect of Ocln knock-down on UVB-induced apoptosis showed a similar effect when using TRAIL.

No primary cytotoxicity (necrotic cell death) was seen in response to TRAIL at early time points (3 h). It was only enhanced in the TRAIL-treated keratinocyte cultures after 20 h, interestingly both in the Ocln-siRNA treated cells and in control cells (Figure 6D), arguing for a secondary cytotoxic effect due to absence of removal of apoptotic cells from cell culture.

### Knock-down of Occludin Shows Only Minor Effects on Keratinocyte Proliferation

Increased proliferation represents a characteristic feature of in-situ and malignant tumors. Therefore, we asked whether Ocln might also contribute to the regulation of keratinocyte proliferation. We did, however, not observe increased proliferation of human keratinocytes in 2D cultures at low Ca^2+^ conditions when treated with Ocln-siRNA, whereas at high Ca^2+^ conditions cell proliferation was slightly reduced. Also in 3D skin models no significant influence of Ocln knock-down on keratinocyte proliferation was evident (data not shown).

## Discussion

Here, we demonstrate that downregulation of Ocln in keratinocytes results in decreased cell-cell adhesion, reduced susceptibility to induction of apoptosis, altered epidermal differentiation, and altered Ca^2+^ homeostasis. This hints for a significance of the Ocln loss proven here in a large panel of cutaneous SCC as compared to SCC precursors and to normal skin. In addition, we demonstrate that changes of Cldn-1, Cldn-4, JAM-A, and ZO-1 can not only be found in SCC but also in precursor lesions and sun-exposed skin.

We identified a frequent loss of Ocln in cutaneous SCC which was not found in its precursors or sun-exposed and non-sun-exposed skin. In line with our results loss or downregulation of Ocln has also been described in most samples of lingual and bronchial SCC [55], [56] suggesting Ocln downregulation as a common characteristic of SCC, irrespective from its origin. In addition, Ocln downregulation was also observed in other cancer entities, e.g. gastric cancer, hepatocellular carcinoma, and breast cancer [17], [18], [19], hinting for a general role in tumorigenesis. In breast cancer cell lines it was shown that overexpression of Ocln can decrease invasiveness and cell motility in vitro and inhibits tumor development and metastasis in mouse experiments in vivo [30]. On the other hand, knock-down of Ocln in breast cancer cell lines resulted in increased invasiveness [18].

Previous reports of cutaneous SCC could not identify such an Ocln loss though the protein was partly found to be restricted to cells with keratinization such as cancer pearls [43], [44]. As we also observed Ocln staining in some of our SCC, this discrepancy might be due to the limited number of cases investigated before. Any staining procedure problems could be largely excluded because we exclusively evaluated tumors with internal positive controls (see materials and methods).

Addressing the question of consequences of reduced Ocln levels in keratinocytes, we investigated epidermal differentiation, markers for EMT, cell-cell adhesion, Ca^2+^-permeability, apoptosis, and proliferation.

We demonstrate that downregulation of Ocln results in downregulation of involucrin and increase of TG1 protein levels in 3D skin models, indicating an influence of Ocln on epidermal differentiation. Several studies described a downregulation of involucrin in poorly differentiated SCC, while it was strongly expressed in highly differentiated SCC [38], [39], [40], [44]. For TG1 increased staining intensity was described in the epidermal part of cutaneous SCC, [41], a frequent (69%) upregulation was also observed in SCC of the lung [57]. However, we do not see a clear correlation of downregulation of Ocln and changes of involucrin and TG1 in our SCC tissues. This reflects that tumor progression is influenced by various internal and external factors that, depending on the tumor-microenvironment, might overrule the effect of downregulation of Ocln and which we cannot mimic in our cell culture system. In addition, we cannot rule out that in SCC alteration of Ocln might not only be a cause but also a consequence of altered differentiation, because the chicken-egg question “TJs – differentiation” has not been solved yet [58]. Therefore the final contribution of Ocln downregulation to altered epidermal differentiation in SCC still has to be clarified.

As a putative underlying mechanism of altered epidermal differentiation we identified an increased paracellular Ca^2+^ permeability in keratinocytes and therefore altered Ca^2+^-homeostasis. Increased permeability might result in an altered tissue Ca^2+^-gradient which is known to influence keratinocyte differentiation [59], [60], [61]. Altered Ca^2+^-homeostasis is also likely to be involved in reduced cell-cell adhesion observed in our Ocln knock-down keratinocytes, because Ca^2+^ is known to be essential for the formation of functional adherence junctions and desmosomes [62], [63], [64]. Reduced cell-cell interaction is a typical hallmark in tumor progression. Involvement of Ocln in cell-cell adhesion was described before for fibroblasts ectopically overexpressing Ocln [50]. Because increased Ca^2+^-permeability in Ocln knock-down cells could also be a secondary effect due to the alteration of other TJ proteins, namely Cldn-2 and 12 which are critical for Ca^2+^-permeability in the intestine [65] we also investigated the mRNA levels of these TJ proteins. There was no change of Cldn-12, but there was a significant decrease of Cldn-2. However, because Cldn-2 normally mediates Ca^2+^-permeability, its downregulation counteracts the increased permeability and might be a compensation mechanism.

Downregulation of Ocln has been described to be involved in EMT. Especially in the context of TGFβ induced EMT recent studies in simple epithelial cells indicate high significance of Ocln as a key regulatory component mediating complex formation of PAR6 together with type I TGFβ receptors. Upon exposure to TGFβ, Par6 is phosphorylated and binds to Smurf1, an E3 ubiquitin ligase, which in turn mediates ubiquitination of RhoA. Loss of Rho is important for the dissolution of TJs and for EMT [66], [67], [68]. However, we did not observe an influence of Ocln knock-down on E-cadherin and vimentin, typical markers for EMT, in our 3D cultures. We also did not observe a correlation of presence/absence of Ocln and the downregulation of E-cadherin or the upregulation of vimentin in our SCC (Table 4). Therefore it is unlikely that Ocln plays a major role in EMT in cutaneous SCC. This might reflect a difference between tumors derived from simple and multilayered epithelia.

We observed a role of Ocln in apoptosis sensitivity of keratinocytes. Thus, reduced apoptotic responses of keratinocytes were seen in response to TRAIL and UVB after Ocln knock-down. TRAIL activates the extrinsic apoptotic pathway, while UVB may induce both, extrinsic and intrinsic apoptotic pathways [69], [70]. In line with our results, a relation of Ocln with extrinsic apoptotic pathways has also been shown in mammary gland cells: Following disruption of TJs by an Ocln-specific peptide, Ocln became associated with the death-inducing signalling complexes (DISC) of death receptors and the extrinsic apoptotic pathway was activated. In mammary gland cells from Ocln knock-out mice this peptide did not induce apoptosis [27]. However, because TRAIL is less effective in differentiated keratinocytes [71], [72], we investigated its effect under low Ca^2+^ conditions. Under these conditions only a low proportion of Ocln is present at the cell-cell borders [73], [74]. Therefore the observed effect of Ocln knock-down in keratinocytes is likely to include an Ocln cell membrane localization-independent effect, while data presented for mammary gland cells suggest that Ocln moves through the plasma membrane to activate the death receptor [27]. In general, the role of Ocln in apoptosis is ambiguous and might depend on the cell type. In HeLa cells over-expression of Ocln enhanced the sensitivity to H_2_O_2_-induced cell death [30], suggesting, in line with our results and the results in mammary gland cells [27], a supportive role of Ocln for the induction of apoptosis. In contrast, in primary hepatocytes from Ocln-deficient mice increased numbers of apoptotic cells were observed and in immortalized cells from this origin apoptosis induced by serum-free conditions was more pronounced than in wild-type cells [28]. Yu et al [29] observed an increase of apoptotic cells in MDCKII cultures after siRNA-mediated Ocln knock-down but this increase was primarily due to the retention of apoptotic cells in the monolayers while overall apoptosis ratio was not affected compared to control cells. We do not observe a significant increase in the number of apoptotic cells in Ocln knock-down cells without treatment with UV or TRAIL compared to control cells. Therefore we conclude that the mechanism in MDCK II cells is different to keratinocytes. We also excluded by our experimental design that the reduced amount of apoptosis observed here in Ocln knock-down cells after induction of apoptosis with TRAIL and UV was due to decreased retention of the apoptotic cells in the monolayers. Interestingly, in our experiments only 40–50% of primary keratinocyte cultures from different donors revealed decreased response to proapoptotic stimuli after Ocln knock-down. Further clarification of the role of Ocln in keratinocyte apoptosis regulation will be a challenging task for future projects.

For Cldn-1, we found downregulation in uppermost and lowermost layers of cutaneous SCC and predominantly absence in the invasive parts, but a complete loss was only found in 9%. This is in agreement with Ouban et al [75] who found Cldn-1 in 91.7% of SCC from different origins. Concerning the downregulation in the uppermost and lowermost layers there was no difference for Cldn-1 between SCC and its precursors, hinting for an early event in skin hyperplasia. This is supported by findings in neoplasia induced by 7,12-dimethylbenz(a)anthracene and 12-O-tetradecanoyl-phorbol-13-acetate treatment of mouse models, which also exhibit downregulation of Cldn-1 in the basal cell layer [76].

Looking for explanations for the common alterations of Cldn-1, Cldn-4 and ZO-1 identified in the various skin tumors, we hypothesized that chronic UV exposure might induce some of these changes. Therefore, we investigated sun-exposed versus non-sun-exposed skin. Indeed, we observed a significant downregulation of Cldn-1 in lower layers and broader localization of Ocln, ZO-1, and Cldn-4 in a high proportion of chronically sun-exposed skin. Our data fit nicely to results reported in an UVB-irradiated murine skin model [77] describing a broader ZO-1 localization throughout the upper epidermis. A broader localization of ZO-1 was also shown in irradiated human skin xenografts and of Ocln in irradiated skin equivalents [78]. Downregulation of Cldn-1 in upper layers which was found in SCC and its precursors, was not observed in sun-exposed skin. Also, opposed to skin tumors, spots of loss for the various proteins were rarely seen in sun-exposed skin. Therefore these events seem to be independent from chronic UV-irradiation.

### Conclusion

In conclusion, we demonstrate a frequent loss of Ocln in cutaneous SCC but not in its precursors. We confirm our hypothesis that Ocln knock-down in keratinocytes is involved in the promotion of tumorigenic features and show reduced susceptibility to TRAIL- and UV-induced apoptosis as well as reduced cell-cell adhesion. This may play a role in SCC tumorigenesis, as well as in other carcinomas, as loss of Ocln is a common feature in tumors. Other changes of TJ proteins were identified in cutaneous SCC as well as in precursor lesions and sun-exposed skin, and may therefore characterize initial steps in tumorigenesis induced by UV-irradiation.
